# Diversity of the Senescence Phenotype of Cancer Cells Treated with Chemotherapeutic Agents

**DOI:** 10.3390/cells8121501

**Published:** 2019-11-23

**Authors:** Agnieszka Bojko, Joanna Czarnecka-Herok, Agata Charzynska, Michal Dabrowski, Ewa Sikora

**Affiliations:** 1 Laboratory of Molecular Bases of Ageing, Nencki Institute of Experimental Biology, Polish Academy of Sciences, Warsaw 02-093, Poland; a.bojko@nencki.gov.pl (A.B.); j.czarnecka@nencki.gov.pl (J.C.-H.); 2 Laboratory of Bioinformatics, Nencki Institute of Experimental Biology, Polish Academy of Sciences, Warsaw 02-093, Poland; a.charzynska@nencki.gov.pl (A.C.); m.dabrowski@nencki.gov.pl (M.D.)

**Keywords:** senescence, senescence markers, cancer, chemotherapy, DNA damage, SASP

## Abstract

It is acknowledged that cancer cells are able to undergo senescence in response to clinically used chemotherapeutics. Moreover, recent years have provided evidence that some drugs can selectively remove senescent cells. Therefore, it is essential to properly identify and characterize senescent cells, especially when it comes to cancer. Senescence was induced in various cancer cell lines (A549, SH-SY-5Y, HCT116, MDA-MB-231, and MCF-7) following treatment with doxorubicin, irinotecan, methotrexate, 5-fluorouracil, oxaliplatin, or paclitaxel. Treatment with tested chemotherapeutics resulted in upregulation of p21 and proliferation arrest without cytotoxicity. A comparative analysis with the use of common senescence markers (i.e., morphology, SA-β-galactosidase, granularity, secretory phenotype, and the level of double-stranded DNA damage) revealed a large diversity in response to the chemotherapeutics used. The strongest senescence inducers were doxorubicin, irinotecan, and methotrexate; paclitaxel had an intermediate effect and oxaliplatin and 5-fluorouracil did not induce senescence. In addition, different susceptibility of cancer cells to senescence was observed. A statistical analysis aimed at finding any relationship between the senescence markers applied did not show clear correlations. Moreover, increased SA-β-gal activity coupled with p21 expression proved not to be an unequivocal senescence marker. This points to a need to simultaneously analyze multiple markers, given their individual limitations.

## 1. Introduction

The rationale behind anticancer strategies is to kill rapidly dividing cancer cells by high doses of drugs or irradiation. However, delayed side effects of anticancer treatment, such as recurrence and secondary cancers, are still serious problems for cancer survivors and clinicians and raise the question concerning the resistance of cancer cells to treatment. One reason for cancer recurrence could be the cytostatic instead of cytotoxic effect of anticancer treatment. It has already been 20 years since anticancer treatment was shown to induce cellular senescence [1] Subsequently, therapy-induced senescence (TIS) has been recognized as an effective way to treat cancer while lessening side effects [2,3]. However, the initial enthusiasm was quickly extinguished by a growing body of evidence proving that senescence of cancer cells can be undesirable due to the propensity of senescent cells to create a procancerogenic microenvironment and the resumption of senescent cell division [4]. A new paradigm of senescent cell therapy has been developed [5] and a new class of drugs—senolytics—has been introduced. Senolytics, which selectively eliminate senescent cells, are currently being introduced to anticancer therapy [6].

Normal cells undergo senescence due to telomere erosion, which is known as replicative senescence [7], or due to oncogenes or stress, resulting in oncogene-induced senescence (OIS) [8] or stress-induced premature senescence (SIPS) [9]. These types of cell senescence are quite rapid and telomere-erosion-independent. Many molecular mechanisms and signaling pathways involved in cell senescence induction and acquisition of the senescence phenotype have been recognized [10]. However, irreversible loss of the ability of cells which were previously proliferation-competent to divide is the most important feature that identifies senescent cells. Thus, cell cycle inhibitors, such as p21 and p16 proteins, along with diminished DNA replication capability, are the main hallmarks of cell senescence [11]. Senescent cells can be arrested in the G1 or G2 phase of the cell cycle. One of the earliest described and the most commonly measured (although not fully specific) markers of senescent cells is the increased activity of a lysosomal enzyme—the senescence-associated β-galactosidase (SA-β-gal) [12]. Other important features of senescent cells include increased number of DNA double-strand breaks (DSBs) measured as γH2AX foci, increased level of key proteins involved in DNA damage response, such as ATM and p53, chromatin remodeling, dysfunctional mitochondria and lysosomes, and increased size and granularity [13]. Senescent cells secrete many factors, including proinflammatory ones, which give rise to the so-called senescence-associated secretory phenotype (SASP) [14].

Cancer senescent cells are generally characterized by the same markers as normal senescent cells. However, in many cancers, the *CDKN2* gene encoding p16 is inactive due to promoter methylation. Accordingly, cancer cell senescence relies mainly on p53/p21 activation, proving that *TP53* is not mutated. In their seminal work, Roninson’s group showed that p53 and p21 act as positive regulators of senescence, but their function is neither sufficient nor absolutely required for this response in tumor cells [1]. We showed that p53-negative colon cancer cells can undergo senescence [15]. As proper identification of cancer senescent cells became an urgent matter due to the fact that they can be more harmful than beneficial, in this study, we aimed to answer the question of whether the process of therapy-induced senescence affects different cells to the same extent. To this end, we have characterized the senescence phenotype of several cancer cell lines treated with different anticancer drugs using a set of common senescence markers. Our results point to a cell type and drug diversity in the cancer cell senescence phenotype.

## 2. Materials and Methods

### 2.1. Reagents

Doxorubicin (D1515), irinotecan hydrochloride (I1406), 5-fluorouracil (F6627), methotrexate (M9929), and paclitaxel (T7402) were purchased from Sigma-Aldrich (Saint Louis, MI, USA). Oxaliplatin (S1224) was purchased from STI (Poznan, Poland).

### 2.2. Culture of Cancer Cells

Human colon HCT116 (CCL-247) cancer cell line was kindly provided by Dr. Bert Vogelstein (Johns Hopkins University, Baltimore, MD, USA). Human non-small-cell lung cancer A549 (CCL-185) cell line was kindly provided by prof. Jolanta Jura (Jagiellonian University, Cracow, Poland), whereas breast cancer MCF-7 (HTB-22) and MDA-MB-231 (HTB-26) cell lines and neuroblastoma SHSY5Y (CRL-2266) cell line were purchased from the American Type Culture Collection (ATCC). Cells were grown under standard conditions (37 °C, 5% CO_2_) in McCoy’s (HCT116), DMEM low glucose (MCF-7) and DMEM high glucose (A549, MDA-MB-231 and SHSY5Y) medium supplemented with 10% fetal bovine serum, 100 units/mL of penicillin, 100 µg/mL of streptomycin, and 25 µg/mL amphotericin B.

To induce senescence, cancer cells were seeded at a density of 10,000/cm^2^ 24 h before treatment with chemotherapeutics. Next, cancer cells were incubated with concentrations of doxorubicin, methotrexate, paclitaxel, 5-fluorouracil, oxaliplatin, or irinotecan that yielded the highest number of SA-β-gal-positive cells without a cytotoxic effect (Table 1). After 24 h, fresh drug-free medium was added. Cells were analyzed in terms of senescence markers three days after drug removal.

### 2.3. Quantification of Senescence-Associated β-galactosidase-Positive Cells

Detection of senescence-associated β-galactosidase (SA-β-gal) activity was performed according to [12]. Briefly, cells were fixed with 2% formaldehyde and 0.2% glutaraldehyde in PBS, washed, and exposed overnight at 37 °C to the solution containing 1 mg/mL 5-bromo-4-chloro-3-indolyl-b-d-galactopyranoside, 5 mM potassium ferrocyanide, 5 mM potassium ferricyanide, 150 mM NaCl, 2 mM MgCl_2_, and 0.1 M phosphate buffer, pH 6.0. Cells (100 or more) were counted under a light Nikon Eclipse 50i microscope (Minato, Tokio, Japan) and the percentage of SA-β-gal-positive cells was calculated.

### 2.4. Measurement of Granularity

Cells were trypsinized and then fixed in 70% ethanol. Next, after washing with 0.05% Tween in PBS, cells were incubated for 5 min with citrate buffer (0.2 M Na_2_HPO_4_ with 0.1 M citric acid pH 7.8), washed with 0.05% Tween in PBS and incubated for 30 min with PI solution (3.8 mM sodium citrate, 500 µg/mL RNAse A, 50 µg/mL PI in PBS). Cell size and granularity were assessed with the use of flow cytometry (FACSCalibur, BD Biosciences, Franklin Lakes, NJ, USA). Data were analyzed with the use of CellQuest^TM^Pro Software (version 6, BD Biosciences, Franklin Lakes, NJ, USA). 10,000 events were collected per sample. All chemicals were purchased from Sigma-Aldrich.

### 2.5. Measurements of Secreted Factors

The secretory phenotype was analyzed by ELISA in culture medium collected on the third day after a 24 h treatment with tested drugs. Experiments were performed according to the protocol provided by the manufacturer (R&D Systems, Minneapolis, MN, USA). Levels of cytokines (IL-6, IL-8, VEGF) in the samples were determined with the use of standard curves and normalized to cell number. Absorbance was measured at 450 nm with the use of a Tecan Sunrise spectrophotometer (Tecan, Männedorf, Switzerland) and absorbance was analyzed with the X-fluor software (version 4, Tecan, Männedorf, Switzerland).

### 2.6. Western Blot Analysis

Living adherent cells were harvested into Laemmli SDS sample lysis buffer, sonicated, and centrifuged at 10,000× *g* for 10 min. Concentration of proteins was estimated by the BCA method; 100 mM DTT and 0.01% bromophenol were added to lysates before separation by SDS-PAGE (8%, 12%, and 15% gels were used). Total protein concentrations were determined using bicinchoninic acid (BCA) protein assay kit, according to the manufacturer’s instructions. The same protein amount (20 to 50 μg) was loaded into each well. Membranes were blocked in 5% nonfat milk dissolved in TBS containing 0.1% Tween-20 for 1 h at room temperature (RT). Then, membranes were probed overnight at 4 °C with antibodies. The primary antibodies used were: anti-ATM (1:500), anti-phospho-ATM Ser1981 (1:500), γH2AX (1:1000) (Abcam, Cambridge, UK); anti-ATR (1:500), anti-phospho-ATR Ser428, anti-phospho-p53 Ser15 (1:500), (Cell Signalling, Leiden, Netherlands); anti-GAPDH (1:50000), anti-H2AX (1:500) (Millipore, Darmstadt, Germany); anti-p53 (1:500) (Santa Cruz Biotechnology, Santa Cruz, CA, USA); anti-p21 (1:500) (Sigma-Aldrich); PARP (1:1000) (BD Biosciences, Franklin Lakes, NJ, USA). The respective proteins were detected after incubation with the horseradish peroxidase-conjugated secondary antibodies (1:2000) (Dako, Glostrup, Denmark), using an ECL system (Thermo Scientific, Rockford, IL, USA; according to the manufacturer’s instructions).

### 2.7. Immunocytochemistry

For detection of 53BP1 and γH2AX foci, and of actin cytoskeleton, cells were grown on cover slides and washed with PBS. Then, cells were fixed with 4% paraformaldehyde. Next, cells were permeabilized with 0.5% or 0.1% Triton X-100 in PBS. Afterwards, cells were blocked with 2% bovine serum albumin (BSA), 1.5% goat serum (GS) in PBS containing 0.1% Triton X-100 for 10 min. After washing, cells were incubated with primary anti-53BP1 antibody (1:500) (Novus Biological, Cambridge, UK) and γH2AX (1:500) (Abcam) or Ki67 (1:500) (Abcam) in blocking buffer for 2 h, and with Alexa 555 or 488 secondary antibody (1:500) (Life Technologies, Eugene, OR, USA), respectively, in PBS for 1 h. Actin cytoskeleton was stained with Alexa Fluor 488 phalloidin (1:50) (Invitrogen, Carlsbad, CA, USA) for 30 min. DNA was stained with Hoechst 33342. 53BP1 and γH2AX foci as well as actin were visualized under a confocal microscope.

### 2.8. Statistical Analysis

Statistical analysis was performed with the use of the STATISTICA 13 program, employing ANOVA, followed by Tukey’s honestly significant difference (HSD) test. A value of *p* < 0.05 was considered statistically significant (*p* < 0.05-*, *p* < 0.01-**, *p* < 0.001-***). All graphs show the mean results from at least three independent experiments. Error bars represent SEM.

## 3. Results

### 3.1. Cell Senescence Screening by SA-β-gal Activity and Cell Morphology 

For our analyses, we used five cell lines, namely, two breast cancer cell lines (MCF-7 and MDA-MB-231 with mutated p53), one lung cancer cell line (A549), one colon cancer cell line (HCT116), and one neuroblastoma (SH-SY-5Y) cell line. The cells were treated for 24 h with one of the three DNA damage inducers, that is, doxorubicin (DOX), irinotecan (IRI), or oxaliplatin (OX); one of the two antimetabolites, that is, methotrexate (MTX) or 5-fluorouracil (5-FU); or with paclitaxel (PTX), which stabilizes microtubules. After treatment, cells were left in the drug-free medium for several days. 

To induce cell senescence, we used drug concentrations that yielded the highest number of SA-β-gal-positive cells without a cytotoxic effect. Table 1 shows the concentrations required for senescence induction. 

**Figure 1:**
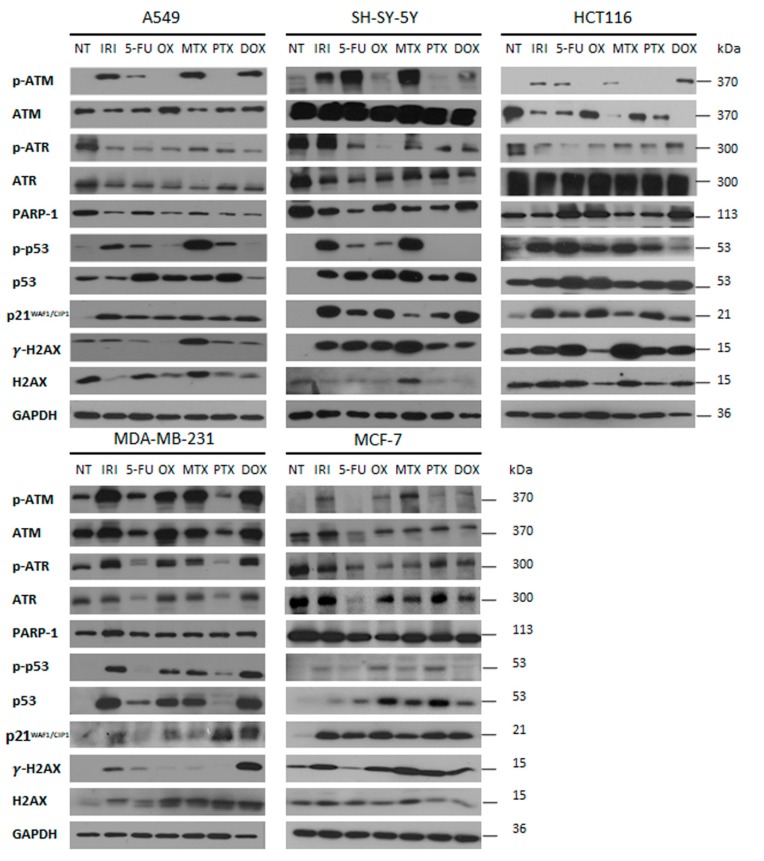
DDR pathway activation and senescence markers at D1+3 analysed by Western blots. Representative Western blot images from three independent experiments (A549 lung cancer cells, SH-SY-5Y neuroblastoma cells, MDA-MB-231 triple-negative breast cancer cells, HCT116 colon cancer cells, MCF-7 breast cancer cells). NT: nontreated cells.

Treatment with drugs at these concentrations, did not cause cell death, as evidenced by the lack of cleaved PARP1. Instead, all cells were characterized by increased p21 level on day 1+3 after treatment (Figure 1). 

As revealed by flow cytometry analysis, only DOX- and IRI-treated cells were arrested in the G2 phase, whereas other drugs arrested cells in the G1 phase, or their influence on the cell cycle was statistically insignificant (data not shown). Ki67 protein is widely used as a proliferation marker as well as a prognostic tool in cancer treatment outcome [16]. Surprisingly, immunofluorescence staining revealed that not all senescent cells were negative for Ki67 (Supplementary Figure S2). Although Ki67 expression decreased in a statistically significant manner after treatment with almost all drugs and in almost all cells (except SH-SY-5Y after MTX and MDA-MB-231 after DOX and IRI), the percentage of Ki67-positive cells remained, in many cases, at a relatively high level (~40%). 

**Figure 2:**
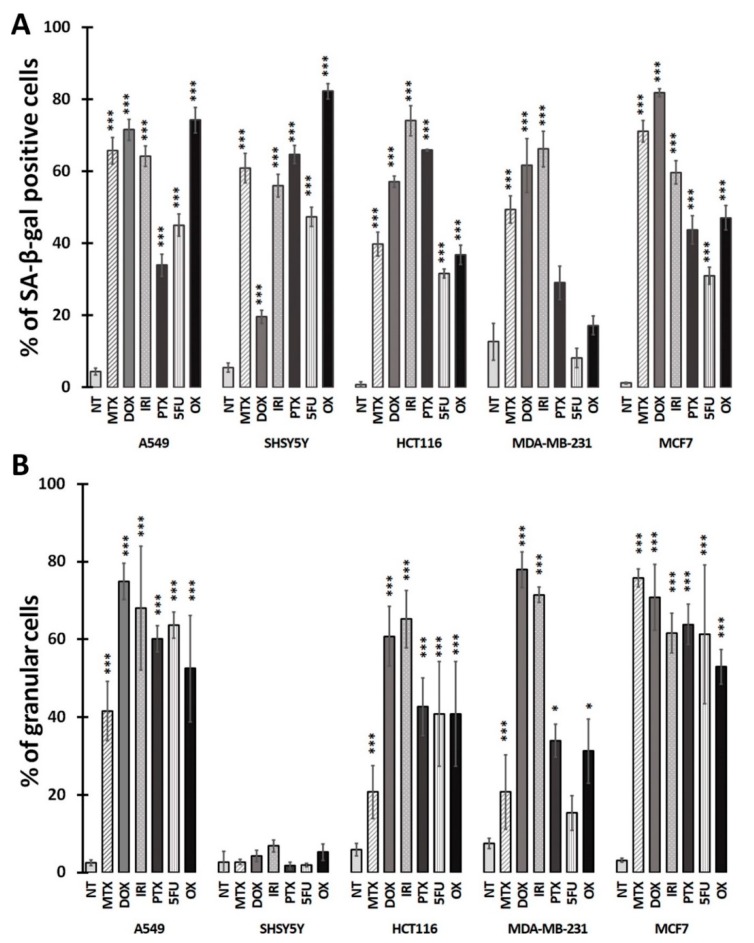
Quantification of SA-β-gal-positive cells (**A**; biochemical activity) and their granularity (**B**; estimated by flow cytometry) on day 3 after treatment (D1+3). NT: nontreated cells. Bars: average of at least three independent experiments, error bars: SEM. Statistical significance (in relation to control): 0.01 < *p* < 0.05, * 0.05 < *p* < 0.001, *** *p* < 0.001. Correlation between elevated activation of SA-β-gal and increased granularity is R = 0.94.

Figure 2A shows the percentage of SA-β-gal-positive cells in all treated cultures (the images of cells stained for SA-β-gal are shown in Supplementary Figure S1). The applied drug concentrations increased significantly the number of SA-β-gal-positive cells among almost all cancer cell types. 

The only exceptions were MDA-MB-231 cells in which PTX, 5-FU, and OX were not able to increase significantly the relative number of SA-β-gal-positive cells. Interestingly, cell granularity, which is another hallmark of cell senescence, shows a different picture than SA-β-gal activity. In three cell line types, namely, A549, HCT116, and MCF-7, all drugs rather uniformly increased the percentage of granular cells to above 40%. Surprisingly, neuroblastoma SH-SY-5Y cells, which in all cases had significantly increased activity of SA-β-gal, did not become granular at all (Figure 2B).

**Figure 3:**
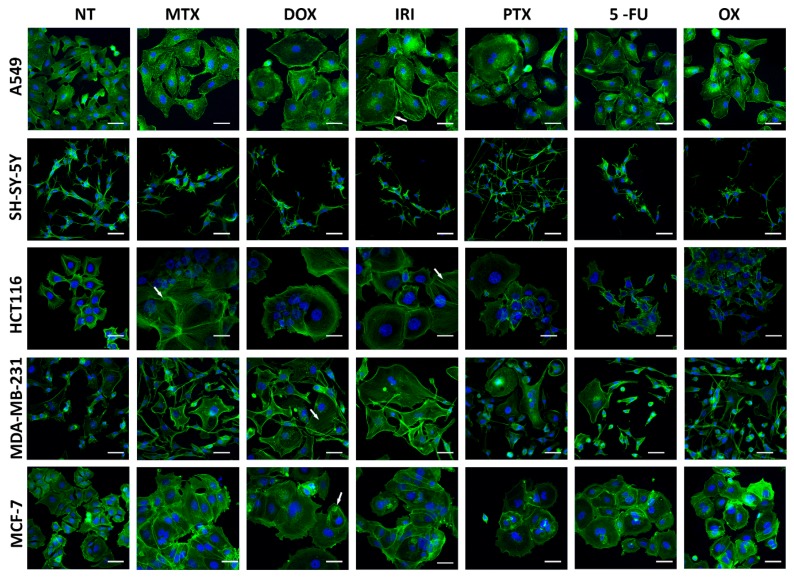
Changes in the morphology of different types of cancer cells upon treatment with chemotherepeutic agents. Representative images of treated cells stained with phalloidin (green), nuclei stained with Hoechst 33342 (blue), day D1+3, arrows indicate stress fibers. Scale bar = 25 µM. NT: nontreated cells.

This was also reflected by cell size as shown in Figure 3. DOX, IRI, and MTX had the strongest effect on cellular morphology, leading to several times size enlargement and flattening of the cells. Senescent cells often were exceeding 50 µM in diameter. Moreover, these drugs caused formation of stress fibers (indicated as arrows in Figure 3), which play a crucial role in cytoskeletal stiffness during senescence. SH-SY-5Y were the only cells which, in spite of having increased SA-β-gal activity, had completely unaltered morphology, both in terms of size and actin cytoskeleton. Other cells, independently of the used drug, were more or less enlarged; however, changes in HCT116 and MCF-7 were the most prominent. 

Altogether, DOX, IRI, and MTX induced morphological changes, characteristic for senescence, simultaneously with a statistically significant increase in cellular granularity and in the number of cells with elevated activity of SA-β-gal in all cell lines, except for SH-SY-5Y. On the contrary, PTX, 5-FU, and OX did not alter cellular morphology, but increased cell granularity and activity of SA-β-gal in a statistically significant manner.

### 3.2. SASP in Therapy-Induced Senescence of Cancer Cells

**Figure 4:**
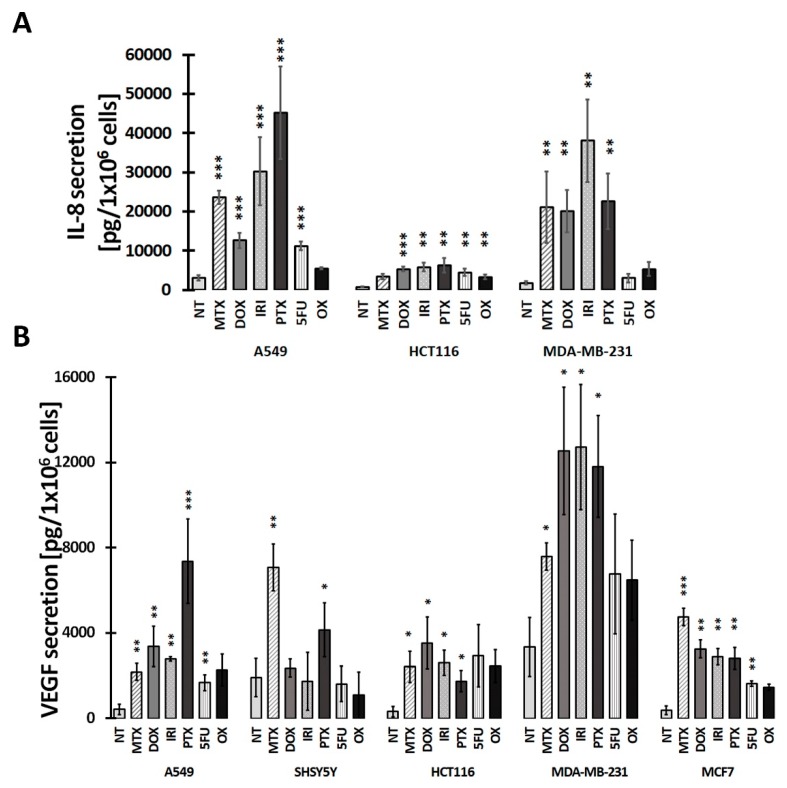
Secretion of inflammatory cytokines IL-8 (**A**) and VEGF (**B**), measured with ELISA. MCF-7 and SH-SY-5Y cells do not secrete IL-8. Bars represent the mean of at least three independent experiments ± SEM. Statistical significance (in relation to control): * 0.01 < *p* < 0.05; ** 0.001 < *p* < 0.01; *** *p* < 0.001, NT: nontreated cells.

Senescent cells remain metabolically active and their secretory activity is the most characteristic feature of cellular senescence [17]. Therefore, we measured the level of the two most abundantly secreted factors, vascular epithelial growth factor (VEGF) and IL-8 chemokine. Figure 4B shows that all types of cancer cells secreted VEGF upon treatment with different drugs, although its level was not always significantly increased in comparison with nontreated cells. IL-8 levels were significantly increased upon treatment with all drugs, with the exception of 5-FU and OX treatment of MDA-MB-231 cells and OX treatment of HCT116 cells (Figure 4A). MDA-MB-231 cells had the highest level of VEGF, independently of the used drug. Even the basal level of this growth factor was high. 

MCF-7 and SH-SY-5Y cells did not secrete IL-8 at all. Two of the analyzed cells, namely, A549 and MDA-MB-231, secreted also IL-6 upon senescence (data not shown). In A549 cells, MTX, DOX, and PTX induced a statistically significant increase in the secretion of IL-6, whereas in MDA-MB-231 cells, all drugs, except for 5-FU and OX, were that effective.

### 3.3. DNA Damage and DNA Damage Response (DDR) in Therapy-Induced Senescence

**Figure 5:**
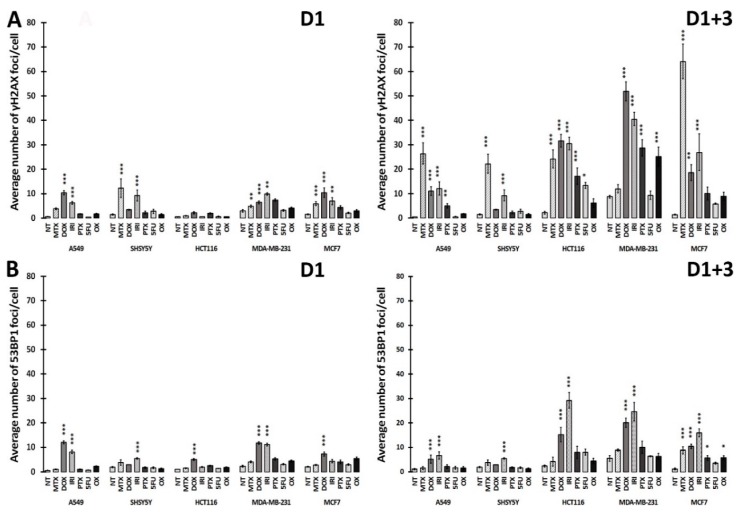
DNA damage response in treated cells. **A**: quantification of the average number of γH2AX foci per cell (left panel D1, right panel D1+3); **B**: quantification of the average number of 53BP1 foci per cell (left panel D1, right panel D1+3). The bars represent mean values of at least three independent experiments ± SEM. Statistical significance (in relation to control): * 0.01 < *p* < 0.05; ** 0.001 < *p* < 0.01; *** *p* < 0.001. NT: nontreated cells.

**Figure 6:**
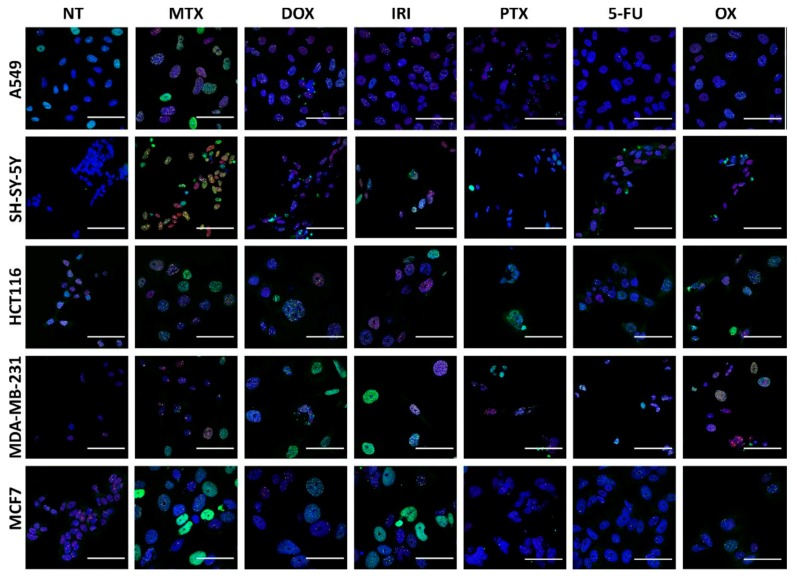
Senescent cancer cells are characterised by enlarged nuclei displaying the highest number of DNA damage foci. Representative images of treated cancer cells at D1+3, immunostained with γH2AX (green) and 53BP1 (red), and nuclei stained with Hoechst 33342 (blue). Scale bar = 50 µM. NT: nontreated cells.

The majority of anticancer drugs are DNA-damaging agents and permanent DNA damage response is the main signaling pathway active in cell senescence [13]. Accordingly, we have checked double-strand breaks (DSBs) in treated cells by analyzing γH2AX DNA damage foci. Phosphorylated H2AX (γH2AX) accumulates immediately in DSBs [18]. To assess the initial steps of DSB repair, we checked the p53-binding protein 1 (53BP1), which promotes NHEJ repair in the G1 cell cycle phase [19] and plays an important role in selecting a DNA repair pathway in G1 and G2/M [20]. Accumulation of this protein was measured on day D1 and day D1+3 in all treated cells and the results of and quantification of γH2AX and 53BP1 foci is presented in Figure 5 (D1 and D1+3) and their immunostaining are shown in Figure 6 (D1+3).

Our results revealed that the number of DSBs, visualized by γH2AX foci, was generally higher on day D1+3 than immediately after drug removal on day D1; however, there were drug and cell differences in the numbers of DNA damage foci. It seems that A549 and SH-SY-5Y cells had less γH2AX foci than other cells. MTX, DOX, and IRI were the strongest inducers of DNA damage in comparison with PTX and OX. Interestingly, MTX seemed to be the most effective DNA-damaging agent in all cells with the exception of MDA-MB-231cells. In turn, these cells acquired a high γH2AX foci number after OX treatment. The number of 53BP1 foci were lower in comparison to the γH2AX foci number, at both analyzed time points. This allows us to draw the conclusion that persistent DNA damage is a rather universal feature of the cancer cell senescence phenotype; however, some diversity in the number of DNA damage foci can be observed depending on the drug and cell type. Proteins from the PIKK family (phosphatidylinositol-3 kinase-like protein kinases), such as ATM (Ataxia-Telangiectasia mutated) and ATR (ATM and Rad3 related), are initiators of DDR signaling. ATM kinase is considered as a pivotal master regulator of cellular senescence, mostly because it phosphorylates the p53 transcriptional factor, which leads to transactivation of the *CDKN1A* gene and p21 protein production [21]. The p21 protein plays multiple roles in senescence initiation and maintenance. Apart from being a cell cycle inhibitor, p21 regulates transcription (positively for senescence genes and negatively for proliferation genes) [22,23] and also blocks apoptosis by different mechanisms, including inhibition of caspase activity [24]. ATR kinase acts mainly via CHK1, blocking the CCNB1 gene expression and cyclin B production, which prevents the entrance into mitosis [25]. Generally, but not always, ATM is activated by DSB, while ATR interacts mainly with single-strand breaks (SSB) [26]. The Western blot technique was used to assess the level of total and phosphorylated proteins involved in DDR, namely, ATM, ATR, p53, γH2AX (Figure 1). Generally, the levels of γH2AX confirmed the results obtained by immunostaining. However, a big diversity in the levels of total and phosphorylated ATM, ATR, and p53, both in control and treated cells, was observed depending on the drug and cell type. The proper assessment of DDR signaling pathways requires a more detailed analysis that would include the kinetics of the process. Nonetheless, in all cases, an increased level of p21, the cell cycle inhibitor and one of the best recognized markers of cell senescence, was observed after treatment. 

**Figure 7:**
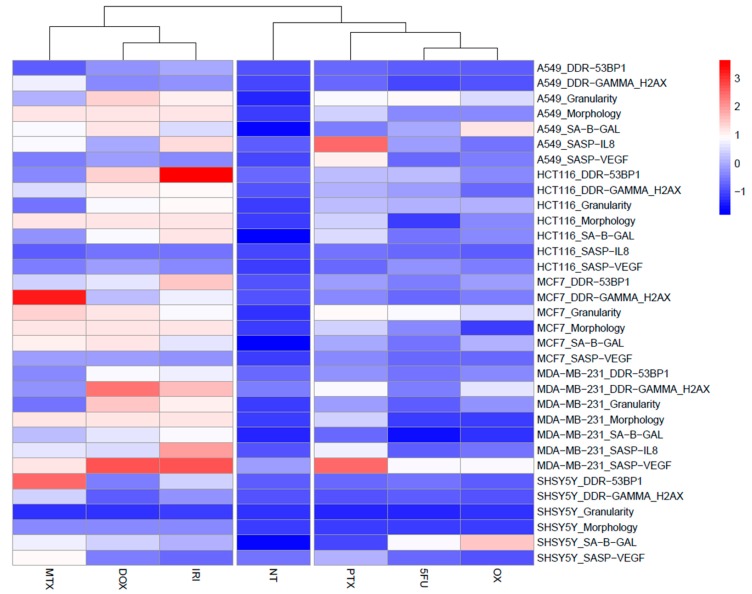
Heatmap showing characteristic features of senescence: senescence morphology (cell size), number of cells with elevated SA-β-gal activity, SASP production, and DDR activation (activation of DDR signaling and number of DNA damage foci) after MTX, DOX, and IRI treatment. Original data points standarized into z-scores. NT: nontreated cells.

**Figure 8:**
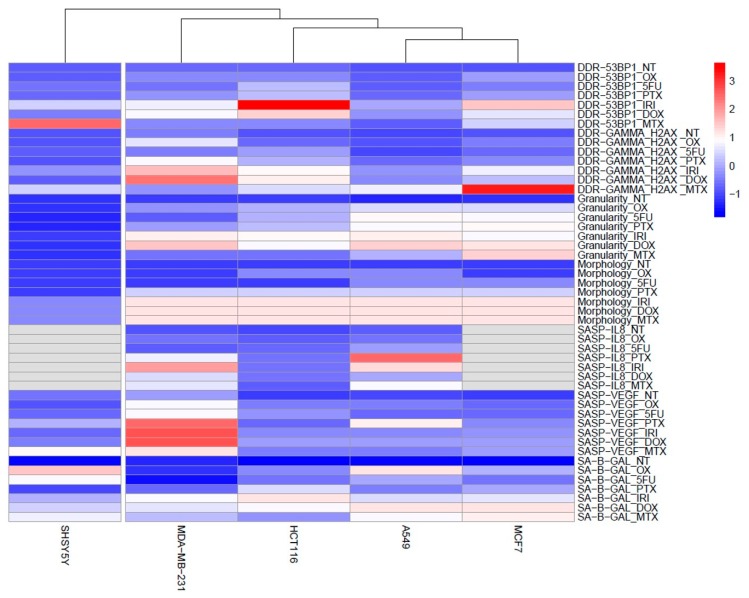
Heatmap showing different susceptibilty of cancer cells to therapy-induced senescence. SH-SY-5Y neuroblastoma cells are the least prone to senesce, whereas MDA-MB-231 breast cancer cells are the most prone ones. Heatmap created with accordance to characteristic features of senescence: senescence morphology (cell size), number of cells with elevated SA-β-gal activity, SASP production, and DDR activation (number of DNA damage foci). Original data points standarized into z-scores. NT: nontreated cells.

Our results showed a big diversity in the quantitative values of different cell senescence markers (number of giant cells, number of granular cells, number of SA-β-gal-positive cells, number of γH2AX-positive and 53BP1-positive cells). Thus, to better visualize how these markers are expressed in various cells treated with different drugs, we have created heatmaps for better comparison of different quantitative values from different assays (Figure 7 and Figure 8). We are discussing this in the Discussion section.

## 4. Discussion

Although therapy-induced senescence of cancer cells has recently become recognized as an undesirable outcome of chemotherapy [27], several molecules that could be used in prosenescence therapy are currently undergoing clinical trials. Moreover, many conventional anticancer treatments induce cancer cell senescence [27,28,29]. Thus, lately, efforts to develop senolytic drugs that can induce death of senescent cells have been intensified [30]. Accordingly, proper characterization of cancer cell senescence is an urgent matter, especially as there is no single universal marker of cell senescence that could be used for unequivocal identification of senescent cells.

The onset of senescence in normal cells is regulated, together or sometimes independently, by two tumor suppressor proteins, p53 and Rb. In response to stimuli that lead to cellular senescence, phosphorylated p53 activates its target genes, including *CDKN1A*, which encodes the cyclin-dependent kinase inhibitor p21. The latter activates Rb through inhibition of a cyclin-dependent kinase complex (E/Cdk2). The hypophosphorylated Rb inhibits transcription of E2F target genes and arrests cells in the S phase. Rb is also activated by another Cdk inhibitor, p16, which acts through the cyclin D/Cdk4,6 complex [31]. Tumor suppressor genes, such as those encoding the Rb, *p53*, and *p16* proteins, crucial for cell senescence, are frequently mutated in cancer cells. Despite this, the hallmarks of cell senescence can be found even in cancer cells lacking active p53/p21 and/or p16/Rb signaling pathways. The best examples are colon cancer p53-negative HCT116 cells, which also have the inactive *CDKN2A* gene encoding the p16 protein but, as we have shown, can still display some features of senescence [15]. P53 is a key protein in DNA damage response (DDR) and many anticancer drugs act as DNA-damaging agents, thus inducing DDR. However, other drugs which do not directly induce DNA damage can also activate DDR due to arresting cells in mitosis [32]. Accordingly, in this study, we have analyzed common markers of cell senescence induced in cancer cells by clinically used drugs, which have different targets in cells.

First, we have shown that all cell types, independently of the drug used, have increased p21 expression, suggesting that, at least temporarily, the cells are arrested in the cell cycle. MDA-MB-231 cells have mutated *TP53*, namely, *TP53R280K*, which results in decreased activation of p53 target genes, including the *CDKN1A*, which encodes p21 [33]. Therefore, in these cells, p21 is induced via different mechanisms. Indeed, it has been documented that p21 level can be increased, for example, via SK2 [34,35]. Treatment with all tested drugs resulted in growth arrest, upregulation of p21 and ATM, and absence of cytotoxicity. However, not in all cell line vs drug combinations was upregulation of p21 connected with ATM activation. This opens a new avenue of eliminating the senescent cancer cells. Crescenzi et al. [36] demonstrated that targeting only p21 or only ATM results in rapid death of senescent cancer cells. Furthermore, we showed previously that inhibition of ATM by KU-55933 blocks DDR and apoptosis induced by etoposide in normal resting T cells. In proliferating leukemic Jurkat cells, KU-55933 had a higher cytotoxic effect [37]. Low dosage of chemotherapeutics induce senescence. Senescent cancer cells could be eliminated by drugs targeting p21, for example, by small molecule inhibitors of p21 used in chemotherapy-resistant kidney cancer [38]. However, it should be stated that prolonged expression of p21 can have detrimental effects leading to senescent cell survival and senescence escape, due to several mechanisms. One of them, proposed by Krizhanonvsky, is noncanonical function of p21, namely, maintaining the viability of DNA-damaged cells by inhibition of the JNK pathway and caspase 3 [39]. Furthermore, chronic p53-independent p21 expression causes genomic instability by deregulating replication licensing [40]. However, increased activity of SA-β-gal coupled with p21 expression is not an adequate senescence marker. Independently of the cell line used, 5-FU and OX upregulated p21 and SA-β-gal, although we cannot consider these cells as senescent ones.

A negative correlation between SA-β-gal activity and Ki67 expression was clearly shown for replicative senescence of normal human fibroblasts [41]. Surprisingly, our results demonstrated that SA-β-gal-positive cells were not always Ki67-negative. High percentage of Ki67-expressing cells after treatment with DOX, IRI, or MTX could be explained by polyploidization during senescence [4,42]. This claim was supported by a relatively high BrdU incorporation rate and increased DNA content (Czarnecka-Herok et al., in preparation). Senescent cancer cells can still express Ki67, therefore Ki67 is not an accurate senescence marker.

When cells are exposed to DNA damage, DSBs (DNA double-strand breaks) generated throughout the genome trigger formation of DDR foci. The vast majority of lesions are repaired. However, those few DSBs that happen to occur at telomeres are not repaired, resulting in persistent DDR and DNA-damage-induced cellular senescence [43]. Many anticancer drugs induce different DNA lesions, which can give rise to DSBs, although there are no data concerning the site of the damage (telomeres versus the whole genome). All agents applied by us (i.e., DOX, MTX, IRI, OX, 5-FU, and PTX, used in the clinic for treatment of different cancer types) can induce DSBs, even if some of them (MTX, 5-FU, and PTX) were introduced to the clinic based on another mechanism of action. 

The primary mechanism of DOX action involves topoisomerase II inhibition and the drug’s ability to intercalate between DNA base pairs, causing breakage of DNA strands. Previously, we have shown that DOX induces DDR in HCT116 cells [15]. There are also other papers showing induction of cancer cell senescence by doxorubicin [44,45]. Surprisingly, in this study, we have not observed ATM and p53 activation upon DOX treatment of HCT116 cells. The reason could be that we have checked the protein levels exclusively on day D1+3. It is reasonable to assume that proper assessment of DDR involvement in cancer cell senescence may require the analysis of the kinetics of the process.

As a structural analog of folic acid, MTX acts through competitively inhibiting dihydrofolate reductase (DHFR). DHFR catalyzes the reduction of dihydrofolate to tetrahydrofolate, which is indispensable for DNA synthesis. There is also evidence that MTX causes DNA damage in cancer cells, such as oxidative damage and DSBs [46]. Previously, we have shown that MTX induces DNA lesions (DSBs) and senescence in colon cancer cells [47]. Irinotecan is a topoisomerase I poison that irreversibly inhibits the enzyme after single-strand cleavage. When the replication fork collides with a poisoned topoisomerase I complex, a DSB with a single free end is generated. OX is a third-generation bifunctional platinum analog that can react with two different nucleophilic centers in DNA. The major lesion generated by oxaliplatin are 1,2-intrastrand linkages between N7 positions of adjacent guanines. 5-FU inhibits thymidylate synthase (TS) and, when cellular deoxythymidine triphosphate is exhausted, DNA replication forks stall. 5-FU can also be incorporated into DNA and DSBs are generated [48]. Paclitaxel is an antimicrotubule agent. It promotes the assembly of microtubules by enhancing the action of tubulin dimers and stabilizing current microtubules while inhibiting their disassembly. Due to the stability of the microtubules, the late G2 phase stops and cell replication is inhibited. Paclitaxel may also distort mitotic spindles, causing the chromosomes to break [49]. It was shown previously that paclitaxel can induce senescence in cancer cells [50]. DNA damage and particularly DSBs induce DDR. In DDR, a plethora of molecules are active in a signaling cascade, from the moment the DNA damage is sensed, until it is translated into outcomes, such as apoptosis, cell cycle arrest, senescence, or DNA repair. The most frequently assessed sensors of DSBs are two proteins: phosphorylated histone H2AX (γH2AX) [18] and the tumor suppressor *P53*-binding protein 1 (53BP1) [51]. γH2AX is the common marker of senescence, while 53BP1 is less frequently used in detecting this process due to the fact that it is involved in nonhomologous end joining (NHEJ) DNA repair process. We have found that the number of γH2AX and 53BP1 foci is generally higher on day D1+3 than day D1. DNA damage sensing is quite rapid; however, in cell senescence, DDR is permanently active as the DNA damage is not repaired. Generally, there is a big diversity in the sensitivity of different types of cells to various DNA-damaging agents. It is of note that DNA damage response could be drug-concentration-dependent. However, accordingly, we have chosen drug concentration that yields the highest number of SA-β-gal-positive cells with no cell death symptoms at a given dose. The rationale for this was that SA-β-gal is the most commonly utilized hallmark of cell senescence. It usually does not give false-negative results, but false-positive results are possible. A serious source of error could be due to high cell density, which was avoided in our experiments. Thus, our results show that practically all drugs are able to activate SA-β-gal in all cells, but the number of SA-β-gal-positive MDA-MB-231 cells was not significantly higher than in the control when the cells were treated with PTX, 5-FU, or OX.

SASP production during senescence could be explained by a classic pathway, namely, induction of DNA damage upon drug treatment, followed by upregulation and activation of ATM. This, in turn, activates the NF-κB signaling pathway, leading to protein translation, and that contributes to secretion of SASP [52]. MTX-, DOX-, and IRI-induced DSBs lead to formation of γH2AX. The latter is indispensable for ATM phosphorylation and activation. A change in the expression level of p53 during the senescence process is likely a critical event, because p53 has antagonistic effects on SASP at least in part by suppressing the activity of p38-MAPK, which is required for NF-κB activation [53]. These findings suggest that establishment of SASP requires coordinated suppression of p53 during the senescence process. Activated p53 transcriptionally induces F-box only protein 22 (Fbxo22), and the SCFF bxo22–lysine-specific demethylase 4A (KDM4A) complex ubiquitylates methylated p53 leading to its degradation. Downregulation of p53 is essential for the induction of senescence-associated secretory phenotype and p16 in senescent cells (late stage of senescence) [54]. This may partially explain why on day D1+3 after DOX treatment, we did not observe p-p53, but strong SASP. Similarly, PTX stimulated strong SASP in all tested cell lines without p53 activation. 

Our results showed a big diversity in the quantitative values of different cell senescence markers, namely, the number of giant cells (morphology), the number of granular cells, the number of SA-β-gal-positive cells, the number of γH2AX-positive and 53BP1-positive cells, and secretion of IL-8 and VEGF. To better visualize how these markers are expressed in various cells treated with different drugs, we have created heatmaps (Figure 7 and Figure 8), which compare quantitative values from different experiments designed for this study. Namely, all data were standardized into z-scores, that is, for each parameter separately, the data points were transformed by subtraction of a sample mean and division by sample standard deviation. This procedure allowed us to compare the values that originated from different statistical distributions; after transformation, the z-scores were close to normal Gaussian distributions.

The first heatmap (Figure 7) shows the efficacy of the applied drugs as inducers of cell senescence. Juxtaposition of senescence markers, after previous data normalization, shows clustering of the results obtained after MTX, DOX, and IRI treatment. Data describing the nontreated cells are connected in hierarchic groups with data obtained after 5-FU and OX treatment. This analysis clearly pinpoints MTX, DOX, and IRI as drugs that induced cellular senescence, whereas 5-FU and OX treatment led to emergence of a phenotype more similar to that of nontreated control cells. We conclude that 5-FU and OX induce growth arrest but not senescence phenotype. Cells treated with those drugs did not proliferate (activation of p53 and p21 upregulation), but were SA-β-gal positive. However, there are neither morphological changes typical for senescence nor DNA damage nor SASP. That implies that increased SA-β-gal activity coupled with upregulation of p21 is not a specific marker for cancer cell senescence. Probably 5-FU and OX induce transient growth arrest, uncoupled from senescence, and after the drugs are metabolized, cells resume proliferation unperturbed (D1+7; data not shown).

The second heatmap (Figure 8) visualizes how the markers are expressed in different cells. The analysis shows that SH-SY-5Y cells, despite having quite high value reflecting DNA damage and activation of SA-β-gal in comparison to other markers (e.g., no altered morphology), can be hardly considered as senescent. SH-SY-5Y are human neuroblastoma cells, which are adrenergic in phenotype but also express dopaminergic markers, and thus are often used as in vitro model of neuronal function and differentiation. We have previously shown that rat cortical neurons in short-term culture acquired high SA-β-gal activity, but we suppose that it could be due to the increased lysosomal biogenesis, hence it is hard to decide whether activity of this enzyme can be considered as a marker of senescence of these cells [55].

Differences in the propensity to undergo cellular senescence probably arise as a result of drug–cell line interaction. Cell lines used by us varied in tissue they were derived from, aggressiveness, and mutations within the signaling machinery. Three cell lines, namely, non-small-cell lung cancer cells (A549; CCL-185), colon cancer cells (HCT116; CCL-247), and neuroblastoma cells (SHSY5Y; CRL-2266), originate from primary tumors. In contrast, breast cancer cells, MCF-7 (HTB-22) and MDA-MB-231 (HTB-26) cells, are both metastatic, derived from pleural effusion. However, MDA-MB-231 cells were described as triple-negative, meaning they lack the EGF, progesterone, and estrogen receptors, and are therefore considered difficult to treat, as most therapies act via blocking one of those receptors. MCF-7 cells express estrogen receptors. Furthermore, all cell lines (except MDA-MB-231 cells) express WT p53 and only SH-SY-5Y cells are positive for p16.

There are different models of therapy-induced senescence that are widely used in examining in vitro that phenomenon. However, independently of the model used (i.e., pulse incubation [42,56], cycles of incubation mimicking chemotherapy regime [57], or constant incubation with the drug [15]), we have shown that cells undergoing senescence express a variety of senescent markers. Moreover, early changes in p21 expression determine whether cells will become senescent or will re-enter the cell cycle after chemotherapy [58]. Given that and according to our results (characteristic morphological changes, increased SASP, persistent DNA damage), evaluating senescent phenotype a few days after chemotherapeutic treatment is justified. Therapy-induced senescence in contrast to replicative senescence is a very rapid process. Therefore, our model allows us to track the cell fate decision and senescence development. Lung, breast, and colon cancers are the most common causes of death among all malignant carcinomas and incidence is increasing worldwide [59]. Therefore, examining whether or not commonly used chemotherapeutics are able to induce TIS seems to be a promising way of fighting cancer. Very recently, a new class of senolytics (cardiac glycosides) was introduced, aiming specifically at lung A549 [60] and colon HCT116 [61] cancer cells, whereas in the case of the neuroblastoma cell line, which is embryonic and neuronal, there is a lack of publication considering TIS.

A statistical analysis aimed at finding any relationship between the senescence markers applied did not show clear correlations. This points to a need to analyze multiple markers, given their individual limitations.

Furthermore, not all clinically used drugs induce the senescent state in cancer cells. The strongest inducers were DOX, IRI, and MTX; the PTX had an intermediate effect, whereas OX and 5-FU did not induce senescence at all. Susceptibility of cancer cells to undergo senescence upon treatment varies, the most prone being the MDA-MB-231 cell line, whereas SH-SY-5Y cells are either resistant to senescence or do not express classic senescence markers at all. Moreover, increased SA-β-gal activity coupled with p21 expression proved not to be an unequivocal senescence marker.

## Figures and Tables

**Table 1:** Chemotherapeutics’ concentrations used to induce senescence.

Drug	Senescence Inducing Concentration				
	A549	SH-SY-5Y	HCT116	MDA-MB-231	MCF-7
**MTX**	32 µM	32 µM	1.25 µM	30 µM	2.5 µM
**DOX**	132 nM	200 nM	100 nM	100 nM	100 nM
**IRI**	21.26 µM	4 µM	2.5 µM	5 µM	5 µM
**PTX**	48 nM	150 nM	7.4 nM	28 nM	56 nM
**5-FU**	50 µM	41.24 µM	50 µM	50 µM	50 µM
**OX**	7.32 µM	7.5 µM	5 µM	10 µM	10 µM

## Supplementary Materials

The following are available online at https://www.mdpi.com/2073-4409/8/12/1501/s1: Figure S1: SA-β-galactosidase activity of treated cells at D1+3. Senescent cells are characterized by accumulation of SA-β-gal within cytoplasm (blue staining), as well as enlarged size and flattened morphology. Scale bar = 100 µm. Figure S2: Percentage of Ki67-positive cells after treatment with tested drugs at D1+3. The bars represent mean values of at least three independent experiments ± SEM. Statistical significance (in relation to control): * 0.01 < *p* < 0.05; ** 0.001 < *p* < 0.01 *** *p* < 0.001. NT: nontreated cells.
