# Supplementary material for: Diversity of the Senescence Phenotype of Cancer Cells Treated with Chemotherapeutic Agents
Source: Cells. 2019 Nov 23;8(12):1501. doi: 10.3390/cells8121501 (PMC6952928; doi:10.3390/cells8121501)
Supplement: Supplementary file 1 [file cells-08-01501-s001.pdf]

Supplementary file

Diversity of the Senescence Phenotype of Cancer Cells Treated with Chemotherapeutic Agents

Agnieszka Bojko [1,†], Joanna Czarnecka-Herok [1,†], Agata Charzynska [2], Michal Dabrowski [2], and Ewa Sikora [1,*]

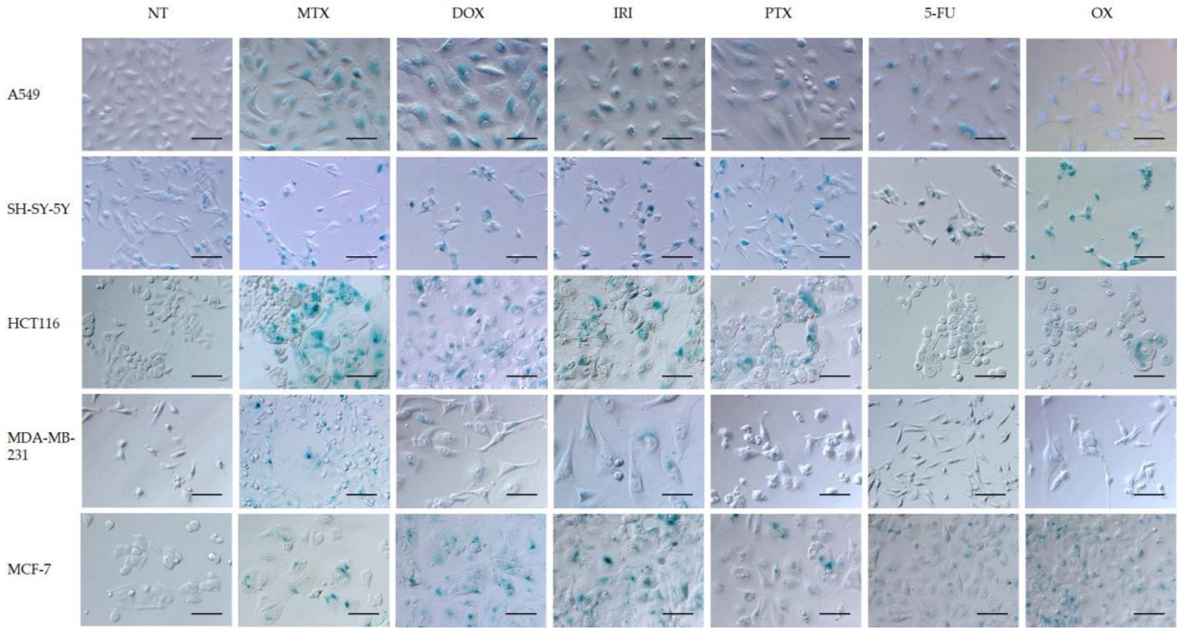

Figure S1. SA-β-galactosidase activity of treated cells at D1+3. Senescent cells are characterized by accumulation of SA-β-gal within cytoplasm (blue staining), as well as enlarged size and flattened morphology. Scale bar = 100 μm.

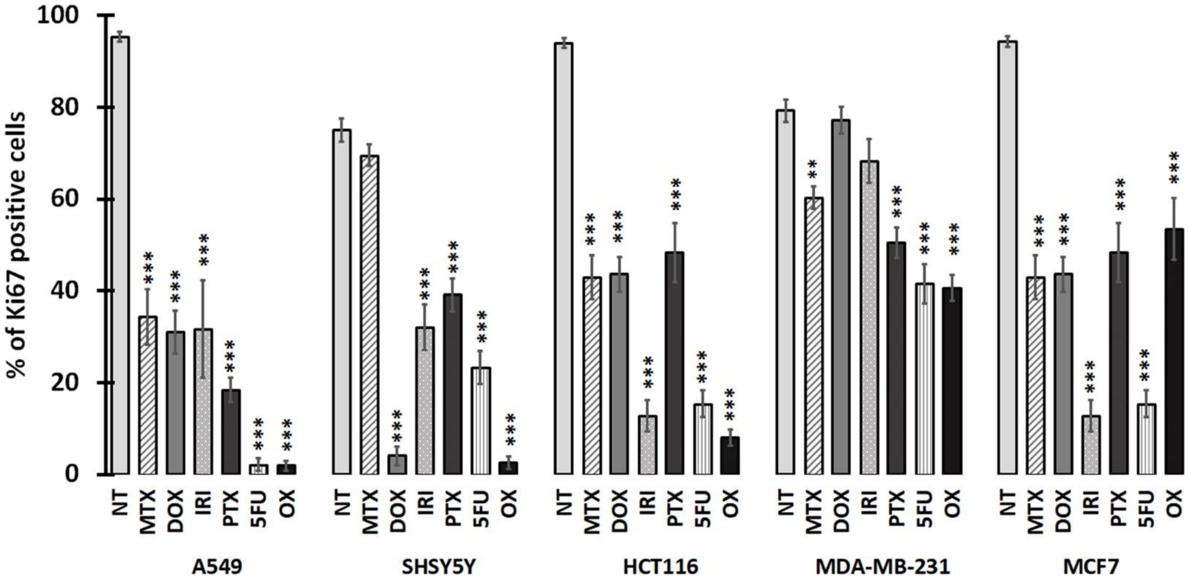

Figure S2. Percentage of Ki67-positive cells after treatment with tested drugs at D1+3. The bars represent mean values of at least three independent experiments ± SEM. Statistical significance (in relation to control): * $0.01 < p < 0.05$; ** $0.001 < p < 0.01$ *** $p < 0.001$. NT: nontreated cells.
